# Correction to: How has COVID-19 affected the treatment of osteoporosis? An IOF-NOF-ESCEO global survey

**DOI:** 10.1007/s00198-021-05905-7

**Published:** 2021-03-09

**Authors:** N.R. Fuggle, A. Singer, C. Gill, A. Patel, A. Medeiros, A.S. Mlotek, D.D. Pierroz, P. Halbout, N.C. Harvey, J.-Y. Reginster, C. Cooper, S.L. Greenspan

**Affiliations:** 1 MRC Lifecourse Epidemiology Unit, University of Southampton, Southampton, UK; 2 Alan Turing Institute, London, UK; 3 Departments of Medicine and Obstetrics and Gynecology, MedStar Georgetown University Hospital, Georgetown University Medical Center, Washington, DC USA; 4 National Osteoporosis Foundation, Arlington, VA USA; 5 International Osteoporosis Foundation, Nyon, Switzerland; 6 WHO Collaborating Center for Public Health Aspects of Musculo-Skeletal Health and Ageing, University of Liège, Liege, Belgium; 7 Biochemistry Department, College of Science, King Saud University, Riyadh, Kingdom of Saudi Arabia; 8 NIHR Musculoskeletal Biomedical Research Unit, University of Oxford, Oxford, UK; 9 Department of Medicine, University of Pittsburgh, Pittsburgh, PA USA

**Correction to: Osteoporosis International (2021)**

10.1007/s00198-020-05793-3

The original version of this article, published on February 8, 2021, contained a mistake. The spelling of Nick C. Harvey’s name was incorrect. The original article has been corrected.

